# 

**Published:** 1899-04-29

**Authors:** 


**Notices of Births, Deaths, and Marriages** are inserted in this column at a charge of 2s. 6d. for an announcement not exceeding four lines.

### NOTICE TO CORRESPONDENTS.

All MS., Letters, Books for Review, and other matters intended for the Editor should be addressed The Editor, "The Hospital," The Hospital Building, 28 & 29, Southampton Street, Strand, W.C.

The Editor cannot undertake to return rejected MS., even when accompanied by stamped directed envelope.

**Notice to Advertisers and Subscribers.**

All Advertisements, Orders for Copies of The Hospital, and Business Communications should be addressed to **THE MANAGER (Not to the Editor)**, The Hospital, The Hospital Building, 28 & 29, Southampton Street, Strand, London, W.C.

The Cost of Subscription, post free, is as follows:—Per week, 2½d.; per quarter, 2s. 8d.; per half-year, 5s. 3d.; per annum, 10s. 6d. Foreign:—Per annum, 15s. 2d., payable, in all cases, in advance.

**NOTICE.—Vol. XXV. of "The Hospital" (October, 1898, to March, 1899), in handsome cloth binding, will shortly be on sale at the Office of this Journal, price 7s. 6d. Cases for binding the Numbers contained in this Volume 1s. 6d. Index to Vol. XXV., 2½d., post free.**

**The Monthly Part for May will be ready May 1st. Price 6d., by post 8d.**

### BOOKS RECEIVED.

H. K. Lewis.

"The Middlesex Hospital Reports of the Medical, Surgical, and Pathological Registrars for 1897."

Wynkoop Hallenbeck Crawford Co., New York.

"Report of the State Charities Aid Association to the State Commission n Lunacy."

### SCRAPS AND GLEANINGS.

The enlargement of the nurses' home of the Bristol Royal Infirmary is soon to be completed. It will provide accommodation for 50 additional beds.

At a special meeting of the governors of the Cardiff Infirmary it was decided that a sub-committee should be appointed to revise the rules of the institution.

The Committee of the Newbury District Hospital are urgently appealing for funds on behalf of their institution, which this year, for the first time since its establishment (now some fifteen years) has been obliged to present an adverse balance sheet.

The receipts at the Royal South Hants Infirmary during last year showed a falling off as compared with the previous year, which was occasioned by the decrease in donations and in workmen's subscriptions as well as in the grant from the Hospital Sunday Fund.

The offer to give £1,000, which was promised last year to the Bristol Royal Infirmary on condition that twelve other persons would each give a like amount, has not yet been taken advantage of, as only one other gentleman has as yet come forward with the required donation.

A resolution was proposed at a special meeting of the governors of Cardiff Infirmary that a deputation should approach the Mayor and County Council of Cardiff with a view to obtaining a grant of not less than £500 a year from the Council to the infirmary towards maintenance expenses, but the resolution was rejected by a majority of ten.

The following rule was adopted at the recent annual court of the trustees and governors of the York County Hospital: "That all cases of accident or extreme urgency be admitted without a subscriber's order, but the committee reserve to themselves the right to make a reasonable charge for the maintenance and attendance of such cases in the event of the person injured proving not to be in necessitous circumstances."

### The Student of Medicine.

### APPOINTMENTS.

W. C. Ambrose, M.R.C.S., L.R.C.P., appointed House Surgeon to the East London Hospital for Children, Shadwell. A. C. Anderson, M.B., C.M.Edin., appointed Assistant House Surgeon to the Taunton and Somerset Hospital. A. E. Bodington, L.S.A., appointed Clinical Assistant to the Chelsea Hospital for Women. F. Brickwell, M.B.Lond., M.R.C.S., L.R.C.P., appointed Junior Assistant Medical Officer in the Cumberland and Westmoreland Asylum, Carlisle. E. Gaylor, L.R.C.P.Edin., L.F.P.S.Glasg., appointed Medical Superintendent Belper Joint District Isolation Hospital. W. H. Gimblett, M.D.Durh., appointed Medical Officer of Health to the Buckhurst Hill Urban District. F. M. Graham, F.R.C.S., L.R.C.P.Edin., appointed Medical Officer for the Whitmore District of the Newcastle-under-Lyme Union. C. A. Hill, M.B., B.C., B.A.Camb., M.R.C.S.Eng., appointed Assistant to the Bacteriologist to the Royal Commission on Sewage Disposal. H. E. Jones, M.R.C.S., L.R.C.P., late Senior Assistant Surgeon, appointed Honorary Surgeon to the Liverpool Eye and Ear Infirmary. M. G. McElligott, D.P.H., L.R.C.P.I., L.R.C.S.I., appointed Deputy Medical Superintendent Belper Joint District Isolation Hospital. J. P. MacLaren, M.A., M.B., C.M., appointed a Clinical Assistant to the Chelsea Hospital for Women. R. C. Martin, M.R.C.S., L.R.C.P., appointed Junior House Surgeon to the Great Northern Central Hospital. S. Tinsley, L.F.P.S.Glasg., appointed Medical Officer for the Huggate District of the Pocklington Union.

